# A total transcriptome profiling method for plasma-derived extracellular vesicles: applications for liquid biopsies

**DOI:** 10.1038/s41598-017-14264-5

**Published:** 2017-10-31

**Authors:** Maria G. Amorim, Renan Valieris, Rodrigo D. Drummond, Melissa P. Pizzi, Vanessa M. Freitas, Rita Sinigaglia-Coimbra, George A. Calin, Renata Pasqualini, Wadih Arap, Israel T. Silva, Emmanuel Dias-Neto, Diana N. Nunes

**Affiliations:** 1 Laboratory of Medical Genomics, A.C.Camargo Cancer Center, São Paulo, SP Brazil; 2 Laboratory of Computational Biology, A.C.Camargo Cancer Center, São Paulo, SP Brazil; 3 Department of Cell and Developmental Biology, Institute of Biomedical Sciences, University of São Paulo, São Paulo, SP Brazil; 4 Electron Microscopy Center, Federal University of São Paulo, São Paulo, SP Brazil; 5 Department of Experimental Therapeutics and Center for RNA Interference & Non Coding RNAs, The University of Texas MD Anderson Cancer Center, Houston, USA; 6 University of New Mexico Comprehensive Cancer Center and Divisions of Hematology/Oncology and Molecular Medicine, Department of Internal Medicine, University of New Mexico School of Medicine, Albuquerque, NM USA; 7 Laboratory of Molecular Immunology, The Rockefeller University, New York, NY USA; 8 Laboratório de Neurociências Alzira Denise Hertzog Silva (LIM27), Instituto de Psiquiatria, FMUSP, São Paulo, SP Brazil

## Abstract

Extracellular vesicles (EVs) are key mediators of intercellular communication. Part of their biological effects can be attributed to the transfer of cargos of diverse types of RNAs, which are promising diagnostic and prognostic biomarkers. EVs found in human biofluids are a valuable source for the development of minimally invasive assays. However, the total transcriptional landscape of EVs is still largely unknown. Here we develop a new method for total transcriptome profiling of plasma-derived EVs by next generation sequencing (NGS) from limited quantities of patient-derived clinical samples, which enables the unbiased characterization of the complete RNA cargo, including both small- and long-RNAs, in a single library preparation step. This approach was applied to RNA extracted from EVs isolated by ultracentrifugation from the plasma of five healthy volunteers. Among the most abundant RNAs identified we found small RNAs such as tRNAs, miRNAs and miscellaneous RNAs, which have largely unknown functions. We also identified protein-coding and long noncoding transcripts, as well as circular RNA species that were also experimentally validated. This method enables, for the first time, the full spectrum of transcriptome data to be obtained from minute patient-derived samples, and will therefore potentially allow the identification of cell-to-cell communication mechanisms and biomarkers.

## Introduction

“Liquid biopsies” are being increasingly recognized as transformative in biology and medicine. Within such context, extracellular vesicles (EVs) such as exosomes and microvesicles are involved in a wide variety of physiological processes and have important roles in cell-to-cell communication during development, as well as in health and diseased states^1,2^. Their capacity to influence the physiology of the recipient cells/tissues is due to the transfer of their cargo of lipids, proteins, and nucleic acids^3,4^, which is produced by their parental cells, selected and loaded into the EVs^5^, and delivered both locally and to distant sites^6,7^. In this sense, the characterization of the full repertoire of EVs-cargo is not only relevant for understanding their potential biological roles, but can also be seen as a source of potential biomarkers of diagnostic and prognostic value in the setting of a wide range of pathological conditions, including cancer, autoimmune or inflammatory, as well as and neurological and infectious diseases.

The key for determining EV content is recovering sufficient amounts of vesicles from patient samples. This challenge is particularly evident in the characterization of EVs present in the peripheral blood of patients, where often only a few milliliters of blood might be available for research investigation, especially in patients with poor clinical conditions and/or advanced disease. Thus far, this practical limitation has hindered a comprehensive analysis of vesicular cargo, and thereby prevented the exploration of the full potential of EVs for clinical applications.

RNA molecules, including microRNAs, long noncoding RNAs and viral RNAs, carried by EVs are amongst the most promising biomarkers for the detection and monitoring of disease^3,8,9^, and may perhaps also be used for monitoring therapeutic response. Notably, recent studies have attempted to profile populations of vesicular RNAs by using next generation sequencing (NGS), to allow the identification of a catalogue of vesicle-derived RNAs (Table 1 **)**. However, most of these studies employed size-selection protocols during NGS library preparation, which has limited the analysis essentially to small RNAs^10–13^. On the other hand, a recent report, has only analyzed RNAs larger than 50 nt, which has essentially excluded molecules such as mature miRNAs^14^. Similarly, amplification steps with oligo-dT primers are also restricted to the study of the polyadenylated fraction of the transcriptome^15^.

**Table 1:** Summary of the recent reports employing RNA sequencing analysis of EVs.

Reference	Sample type	Number of EVs samples	EVs isolation methodology	rRNA depletion	Input	RNA-Seq methodology	Raw reads (sample)	Mapped reads (sample)	rRNA	# Molecules identified	Major categories of molecules identified	Technical validation
Nolte-’t Hoen *et al*., 2012^11^	CCM from mouse DC-T cell co-culture	1	Ultracentrifugation	No	450 ml CCM	Small RNA (15–70 nt) size-selection on native 6% gradient PAGE gel Sequencing on Illumina (single-end 35 cycles)	28.8 M	27.5 M	~30%	NA	tRNA repeat, Simple repeat LINE, rRNA, vRNA, Protein coding, SRP-RNA, Y-RNA	qPCR for miR-29a, miR-155, miR-191, Y-RNA, SRP-RNA
Huang *et al*.^10^	Human plasma	3 = 14 libraries	ExoQuick	No	250 μl plasma/2ng RNA	Small RNA (20–40 nt) size-selection on native 5% acrylamide gel Sequencing on Illumina HiSeq. 2000 (single-end)	7.27 M	4.04 M	9.16%	593 miRNAs	miRNA (76.20% of all mappable reads), ribosomal RNA (9.16%), long noncoding RNA (3.36%), piwi-interacting RNA (1.31%), transfer RNA (1.24%), small nuclear RNA (0.18%), and small nucleolar RNA (0.01%); fragments of coding sequence (1.36%), 5′ untranslated region (0.21%), and 3′ untranslated region (0.54%)	qPCR for miR-92a-3p, miR-191-3p, miR-26b-5p
Jenjaroenpun *et al*.^16^	CCM from MDA-MB-231 and MDA-MB-436, human metastatic breast cancer cell lines	2	Ultracentrifugation	RiboMinus (Thermo) did not work, authors argue due to fragmented rRNA	48 h CCM/ 200ng RNA	Whole transcriptome library with RNAse III fragmentation Considered only reads ≥ 20 bp Sequencing on Ion Torrent PGM	3.3 M	3 M	97%	16,086 transcripts (RPKM ≥ 1)	small nucleolar RNA, small nuclear RNA, Mt_tRNA, microRNA	qPCR for GAPDH, EEF1A1, FTH1, FTL, RAB13, RPPH1, RPL28
Schageman *et al*.^17^	CCM from HeLa cells and human serum	3 = 7 libraries	Total exosome RNA and protein isolation kit (Thermo Fisher)	No	4 ml serum/~2ng RNA	Small RNA bead-based Sequencing on Ion Torrent PGM Considered only reads > 17 bp	5–6 M	90–98%	~15–40%	NA	mRNA, rRNA, tRNA, miRNA, ncRNA	serum EVs - qPCR for has-mir-1281, has-mir-4257, has-mir-451
Li *et al*.^18^	Human serum and urine	4 = 8 libraries	Total exosome RNA and protein isolation kit (Thermo Fisher)	No	4 ml serum/10 ml urine/ ~2ng RNA	Small RNA bead-based Sequencing on Ion Torrent PGM	5–6 M	90–98%	serum: 5–30% urine: 30–60%	NA	miRNA, rRNA, tRNA, mRNA, piRNA	NA
Yuan *et al*.^12^	Human plasma	192	ExoQuick	No	2–10ng RNA	Small RNA (20–40 nt) size-selection on native 5% acrylamide gel Sequencing on Illumina HiSeq. 2000	12.6 M	5.4 M	0.7%	3,387 RNAs	miRNAs (~40.4%), piwiRNAs (~40.0%), pseudo-genes (~3.7%), lncRNAs (~2.4%), tRNAs (~2.1%), and mRNAs (~2.1%)	No
Lefebvre *et al*.^14^	CCM from A431 epidermoid carcinoma and HepG2 hepatocellular carcinoma cell lines	2	Ultracentrifugation	No	80 ml CCM/ 50ng RNA	No size selection was performed - only analysed RNAs > 50 nt Sequencing on Illumina HiSeq. 2000	NA	6.88 M	91.19%	7,361 RNAs (FPKM ≥ 5)	miscRNA, mRNA, lincRNA, snRNA, snoRNA, antisense	qPCR for TPT1, PABPC1, ATF4, PTBP1, HDGF, G3BP1,BRAF
San Lucas *et al*.^15^	Human pleural effusion and plasma	3	Ultracentrifugation	No	800 ml pleural effusion 15 ml plasma	mRNA amplification using oligo dT primers Sequencing on Illumina HiSeq. 2500	NA	498 M	NA	NA	Protein coding	No
Quek *et al*.^13^	CCM from GT1–7 mouse hypothalamic neuronal cell lines	5 = 23 libraries (4 gradient fractions, 1 UCexo)	Ultracentrifugation and Optiprep gradient	No	96 h CCM/ 20ng RNA	Small RNA bead-based Sequencing on Ion Torrent PGM	NA	1 M	0.54%	515 small RNAs	fragments of tRNA (range 41.6–67.0%), fragments of RNA repeat elements (~42.26%), miRNA (range 0.48–2.11%), protein-coding mRNA (range 0.49–1.82%), piRNA (range 0.46–1.71%), snRNA (range 0.11–0.23.%), snoRNA (range 0.04–0.10%), rRNA (range 0.20–0.88%)	qPCR and dPCR for let-7b and miR-342-3p

M: million, nt: nucleotides, bp: base pairs, CCM: cell conditioned medium, EVs: extracellular vesicles, NA: information not available, RPKM: reads per kilobase per million mapped reads, FPKM: fragments per kilobase of transcript per million mapped reads, RPM: reads per million, qPCR: quantitative real time PCR, UCexo: exosomes from ultracentrifugation, dPCR: digital PCR.

In order to address such technical limitations, here we have developed a streamlined new methodology of total transcriptome profiling of EVs by NGS, which includes the simultaneous analysis of both small and long RNAs. In order to do so, total RNAs were fragmented by enzymatic digestion and technical modifications for library construction were adopted, leading to an increase in the total number of molecules, which subsequently led to reduced adaptor-dimers. Moreover, by not excluding RNAs of any particular size, our method allows an unbiased characterization of the total transcriptional landscape of EVs, which still remains largely unknown.

## Material and Methods

### Blood collection, EV isolation, and RNA extraction

This study was approved by the research ethics committee of A.C. Camargo Cancer Center (ACCCC; protocol 1554/11). Blood samples were collected in BD Vacutainer tubes with Acid Citrate Dextrose (ACD) solution, from healthy volunteer individuals participating in the cancer prevention campaign at the ACCCC (specifically, a total of five women with no mammogram evidence of breast cancer), after signing an Institutional Review Board-approved written informed consent form. Plasma samples were separated after two centrifugations at 2,500 × g for 15 min and stored at −80 °C. EVs were isolated from 2.5 ml of plasma by ultracentrifugation, as described^19^, but no filtering steps were performed, to allow a broader view of the circulating EVs transcriptome. After the centrifugations at 2,000 × g for 30 min and 12,000 × g for 45 min (Eppendorf centrifuge 5810R, fixed-angle rotor F-34-6-38), pellets were discarded to eliminate cellular debris and apoptotic bodies. Supernatants were then centrifuged at 110,000 × g for 120 min and the resulting EV-containing pellets were washed once in phosphate-buffered saline (PBS) and centrifuged again at 110,000 × g for 70 min (Beckman ultracentrifuge Optima L-90K, swinging rotor SW-41Ti, polypropylene tubes Beckman 331372). PBS was discarded and the final EV-pellet was finally resuspended in 350 μl of lysis solution and RNA was extracted by using spin columns (Total RNA Purification Kit – Norgen Biotek, Canada). As large quantities of RNA were needed for optimization of the RNA fragmentation step, RNA derived from blood buffy coat was used for fragmentation-standardization purposes. For this purpose, blood was collected in two Vacutainer tubes (Becton Dickinson, USA) containing di-potassium ethylenediaminetetraacetic acid (K_2_ EDTA); plasma was separated after two centrifugations at 2,500 × g for 15 min, the buffy coat (100 μl) was transferred to a fresh Eppendorf tube followed by the addition of 350 μl of lysis solution and RNAs were extracted (total of four separate extractions) by using spin columns (Total RNA Purification Kit – Norgen Biotek, Canada).

### Electron microscopy

EVs isolated from 4.5 ml of plasma (as described above) and resuspended in at least 50 μl PBS after ultracentrifugation, were washed, pelleted again and finally resuspended in 50 μl of PBS containing 4% methanol-free formaldehyde. Whole-mount negative staining of EVs was performed as described^19^. Briefly, a nickel formvar/carbon-coated grid (Electron Microscopy Sciences, USA) was floated on top of a drop of 15 μl fixed-EVs sample for 20 min, and then washed three times with sodium cacodylate buffer, fixed with PBS containing 1% glutaraldehyde for 5 min, washed seven times with double-distilled water, contrasted with uranyl oxalate for 5 min, embedded with a admixture of ice-cold uranyl acetate/methyl cellulose (1:9 v/v) for 10 min, then dried and analyzed in a JEM-1010 electron microscope (JEOL, Tokyo, Japan) at 80 kV.

### NanoSight

EVs isolated from 200 μl of plasma (as described above) were resuspended in 500 μl of filtered PBS and evaluated by a NanoSight LM10 (Malvern, UK). Images were acquired for 60 sec (triplicates for each sample) with the following parameters: camera shutter − 1495; camera gain − 512; detection threshold − 10.

### Western blot

For protein extraction, EV-pellets (obtained after ultracentrifugation of a pool of four plasma samples, total 6 ml) were resuspended in 100 μl lysis buffer containing 300 mM NaCl, 50 mM Tris pH 7.4, 0.5% NP-40 and anti-proteases cocktail (Roche, Indianapolis, IN, USA). Protein concentrations were determined by the Bradford assay (Bio-Rad, Hercules, CA, USA), and 15 μg total protein was submitted to sodium dodecyl sulfate polyacrylamide gel electrophoresis (SDS-PAGE). Ponceau staining was performed before blocking with 5% milk. Primary commercially available antibodies used were CD63 1:1,000 (CBL553, Millipore, Billerica, MA, United States), RAB27B 1:250 (HPA019849, Sigma-Aldrich), Flotillin 1:500 (ab41927, Abcam, Cambridge, MA, USA), HSP70 1:1,000 (EXOAB-Hsp70A-1, System Biosciences, Mountain View, CA, USA), TSG101 1:500 (ab4A10, Abcam, Cambridge, MA, USA) and RAB7A 1:1,000 (ab50533, Abcam, Cambridge, MA, USA).

### RNA fragmentation optimization

Each aliquot of total RNA, extracted from buffy-coat as described above, was treated with 2U DNAse (TURBO DNA-free kit – Thermo Fisher Scientific, USA), pooled, purified and concentrated with RNeasy MinElute Clean-up Kit – Qiagen, USA). RNA was quantified by NanoDrop and individual 500ng RNA aliquots (in 8 μl of RNase-free water) were fragmented at 37 °C with 1 μl RNAse III and 1 μl 10X RNAse III Reaction Buffer (both from Thermo Fisher, USA), for the following time points: 15, 30, 45, 75, 105, 135, 180, 240, 300, 360 and 420 min. After each time point, 20 μl of RNase-free water were added to each RNA-aliquot, which was further purified with magnetic Nucleic Acid Binding Beads (Thermo Fisher, USA). The size-range of each aliquot of fragmented RNA was visualized with Bioanalyzer Pico and Small RNA chips (Agilent, USA).

### Library construction and sequencing

Whole transcriptome libraries were constructed by using reagents provided in the Ion Total RNA-Seq Kit v2 (Thermo Fisher, USA), following the user guide of the Total Exosome RNA and Protein Isolation Kit (Thermo Fisher, USA), but including several protocol modifications, marked with an asterisk (*). Due to the limited yield, the whole eluate volume of 50 μl was vacuum concentrated to 16 μl, to allow all the extracted mass of RNA to be used. The total RNA volume was then fragmented by RNAse III at 37 °C for 180 min*, following the standardization described above. This step served to increase the number of RNA molecules available to hybridize with adaptors, minimizing the formation of adaptor dimers and thereby allowing the simultaneous analysis of the whole transcriptome, including all size-classes of RNAs, in a one-step library preparation protocol. The resulting RNA molecules were purified with magnetic Nucleic Acid Binding Beads* (following the recommendation in the Ion Total RNA-Seq Kit v2 protocol for Whole Transcriptome libraries, as opposed to spin columns, in order to minimize RNA loss), and hybridization to the Ion Adaptor Mix v2 was performed at 65 °C for 30 min* (the incubation time was extended to allow more time for the RNA molecules to hybridize to the adaptors), followed by ligation at 16 °C for 16 h. Templates were denatured at 75 °C for 15 min* (the incubation time and temperature were increased to assure a total denaturation of RNA secondary structures), followed by cDNA synthesis at 42 °C for 30 min. After bead-purification, the cDNAs were amplified and barcodes added to each sample by PCR* (an extra 2 PCR cycles were performed and extension steps were removed to disfavor the amplification of longer fragments). After bead-purification of amplified libraries, molarities of final libraries were determined by quantification on a Bioanalyzer instrument using the High Sensitivity DNA Kit (Agilent, USA). Libraries were diluted to the recommended concentration for emulsion PCR on OneTouch2 instrument (Thermo Fisher, USA). Enriched samples were finally deposited on semiconductor chips (P1) for sequencing on the Ion Torrent Proton platform.

### Bioinformatics analysis

**Figure 1:**
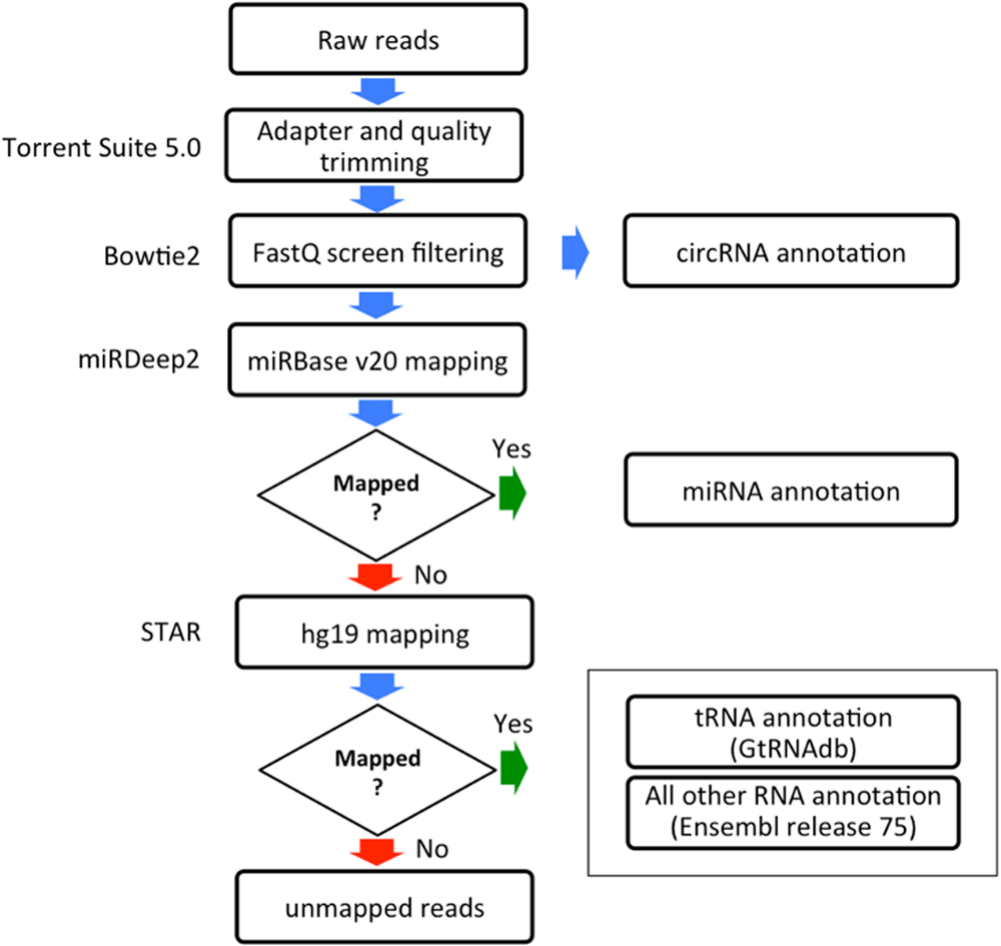
Scheme of the bioinformatics analysis pipeline.

The bioinformatics pipeline started with the filtering of low quality reads (Torrent Suite 5.0 trims the reads to achieve >Phred 15 in a sliding-window of 30 bases). The annotation of the total transcriptome consisted of an initial step with the FastQ Screen^20^ to remove rRNAs, repetitive, as well as other non-human DNA elements. miRDeep2 software^21^ was used to annotate miRNAs in miRBase v20 and unmapped reads were aligned against hg19 genome reference, by using the STAR aligner^22^. tRNAs were annotated by using GtRNAdb database, followed by Ensembl Homo_sapiens.GRCh37.73 for all other transcript annotations. Reads with low mapping scores (Phred < 20, indicating alignment to multiple genomic loci) were removed. To filter out genes with non-uniform reads distribution along the exons, likely to be derived from spurious mapping, we adopted an approach based on Gini normalization coefficient^23^ as a measure for the mRNA coverage variation (mcv). For this purpose, the mRNA for each RefSeq gene was divided into 10 sub-regions designated as ‘bins’. In each such bin, we counted the number of aligned reads and applied to it the Gini coefficient to calculate the mcv-score. The genes with mcv-scores <0.7 were retained for further analysis. The circRNAs prediction was performed with the algorithm find_circ, following the default settings as described^24^ (Fig. 1 **)**.

### qRT-PCR validation

Quantitative RT-PCR (qRT-PCR) was performed to confirm the intra-vesicular origin of selected molecules, as well as the quantification inferred from NGS-data. For this purpose, EVs pellets derived from 0.5 ml of plasma, were isolated by ultracentrifugation as described above, resuspended in PBS and treated with RNase-A (Thermo Fisher, USA) in 0.5 μg/μl final concentration at 37 °C for 20 min. After treatment, RNase-A was inactivated with 1U/μl of RNase Inhibitor (New England Biolabs, USA) at room temperature (RT) and RNA was extracted as described above. To verify the RNase-A activity, a parallel preparation was performed with the same samples wherein the EVs pellet was resuspended in lysis buffer (100 mM Tris, 5 mM EDTA, 0.2% SDS, 0.2 M NaCl, 0.1 ml/ml proteinase K), followed by proteinase-K inactivation at 90 °C for 5 min and RNase-A incubation and treatment with RNase inhibitor and RNA extraction as above. cDNAs were synthesized with miScript II RT Kit (Qiagen, USA) in 20 μl reactions containing 12 μl of RNA, 4 μl of 5X miScript HiSpec Buffer, 2 μl of 10X miScript Nucleics Mix and 2 μl of miScript Reverse Transcriptase Mix, which was incubated at 37 °C for 60 min and 95 °C for 5 min. cDNAs were pre-amplified with miScript PreAMP PCR Kit (Qiagen, USA) in 25 μl reaction containing: 5 μl of cDNA diluted 1:5, 5 μl of miScript PreAMP Buffer, 2 μl of HotStarTaq DNA Polymerase, 5 μl of pool of miRNA assays of interest, 7 μl of nuclease-free water, and 1 μl of miScript PreAMP Universal Primer. Reactions were incubated at 95 °C for 15 min, followed by 12 cycles of 94 °C for 30 sec and 60 °C for 3 min. Pre-amplified cDNAs were analyzed in a 7500 Fast Real-Time PCR System (Thermo Fisher, USA) with miScript SYBR Green PCR Kit (Qiagen, USA) in 10 μl reaction containing 5 μl of 2X QuantiTect SYBR Green PCR Master Mix, 1 μl of 10X miScript Universal Primer, 1 μl of 10X miScript primer assay (miR-223-3p: MS00003871, let-7g-5p: MS00008337), 2 μl of nuclease-free water, and 1 μl of pre-amplified cDNA diluted 1:20.

### circRNA validation

Validation of a representative circular RNA, transcribed from the *CORO1C* gene, was performed by PCR amplification by using outward primers, followed by Sanger sequencing (Suppl. Figure 1). For this purpose, after RNA extraction as described above, cDNA was synthesized by using the SuperScript III First-Strand Synthesis System for RT-PCR (Thermo Fisher, USA). The 20 μl reaction consisted of 8 μl RNA, 50ng of random hexamers, 1 μl of 10 mM dNTP mix, which was incubated at 65 °C for 5 min, 4 °C for 1 min, followed by the addition of the reagents: 2 μl 10X RT buffer, 4 μl 25 mM MgCl2, 2 μl 0.1 M DTT, 1 μl RNaseOUT (40U/μl) and 1 μl SuperScript III RT (200U/μl). The reactions were incubated at 25 °C for 10 min, 50 °C for 50 min, 85 °C for 5 min, 4 °C for 1 min. Finally, 1 μl of RNase H (2U/μl) was added to the reactions and incubated at 37 °C for 20 min.

The PCR primers were designed in such a way to amplify either the linear (inward facing primers, LF and LR) or circular isoforms (outward facing primers, CF and CR). The sequences (5′−3′) of primers used are:

LF: GCTGCTGAATGTGTTGAGGT and LR: GCTGCCTTTCTATGACCCTG; CF: ACCTCAACACATTCAGCAGC and CR: CAGGGTCATAGAAAGGCAGC.

The 15 μl PCR consisted of: 7.5 μl 2X GoTaq Green Master Mix (Promega, USA), 2 μl F primer at 2.5pmol/μl, 2 μl R primer at 2.5 pmol/μl, 2.5 μl nuclease- free water and 1 μl cDNA. PCR cycling conditions were: 95 °C for 2 min followed by 40 cycles at 95 °C for 1 min, 60 °C for 30 sec, 72 °C for 30 sec and final extension at 72 °C for 5 min. Afterwards 10 μl of each PCR product was evaluated on 8% polyacrylamide gel stained with silver^25^ and 5 μl was purified with ExoSAP-IT (Affymetrix, USA) and sequenced by the Sanger method.

### Data availability

All annotation of mapped reads generated or analysed during this study are included in this published article (and its Supplementary Information files). Raw data are available from the corresponding author on reasonable request.

## Results

### EVs characterization

**Figure 2:**
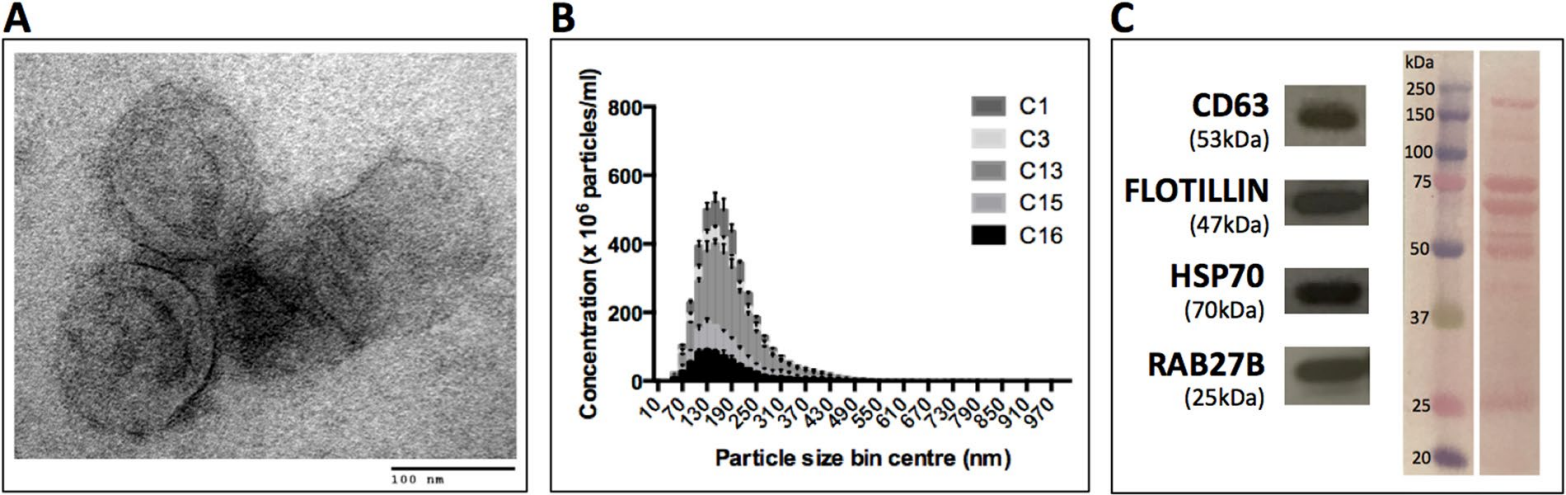
Characterization of EVs isolated from plasma by ultracentrifugation. (**A**) Whole-mount transmission electron micrograph of EVs displaying the characteristic “cup-shaped” morphology. (**B**) NanoSight quantification plot of EVs concentration in function of size shows that the majority of EVs are less than 250 nm in the healthy control samples (n = 5). (**C**) Western blot of the vesicle-associated markers CD63, FLOTILLIN, HSP70 and RAB27B from a pool of plasma samples (n = 4) and corresponding Ponceau staining of membrane before blocking.

We used blood samples isolated from healthy women (n = 5) and isolated EVs as described^19^. To certify that we were indeed working with *bona fide* EVs, the isolated particles were first validated by transmission electron microscopy (TEM), Nanoparticle Tracking Analysis (NTA) and Western blotting. TEM revealed the characteristic “cup-shaped” EVs with morphology, which has been previously described^19^ and sizes typical of exosomes (Fig. 2A), and NTA showed the expected size-range (174–193 nm, and an average size of 185 nm - considering averages from the five plasma samples) an average concentration of 8 × 10^8^ particles/ml (range 4–18 × 10^8^ particles/ml), consistent with published reports^10,15^. Most EVs were smaller than 250 nm, consistent with a heterogeneous mixture of exosomes and microvesicles (Fig. 2B). Western blot analysis of EV-pellet obtained from a pool of plasma samples confirmed the expression of a panel (n = 6) of standard vesicular protein markers: CD63, FLOTILLIN, HSP70, RAB27B (Fig. 2C) and TSG101, RAB7A (Suppl. Figure 3).

### Optimization of RNA input for NGS library-construction

**Figure 3:**
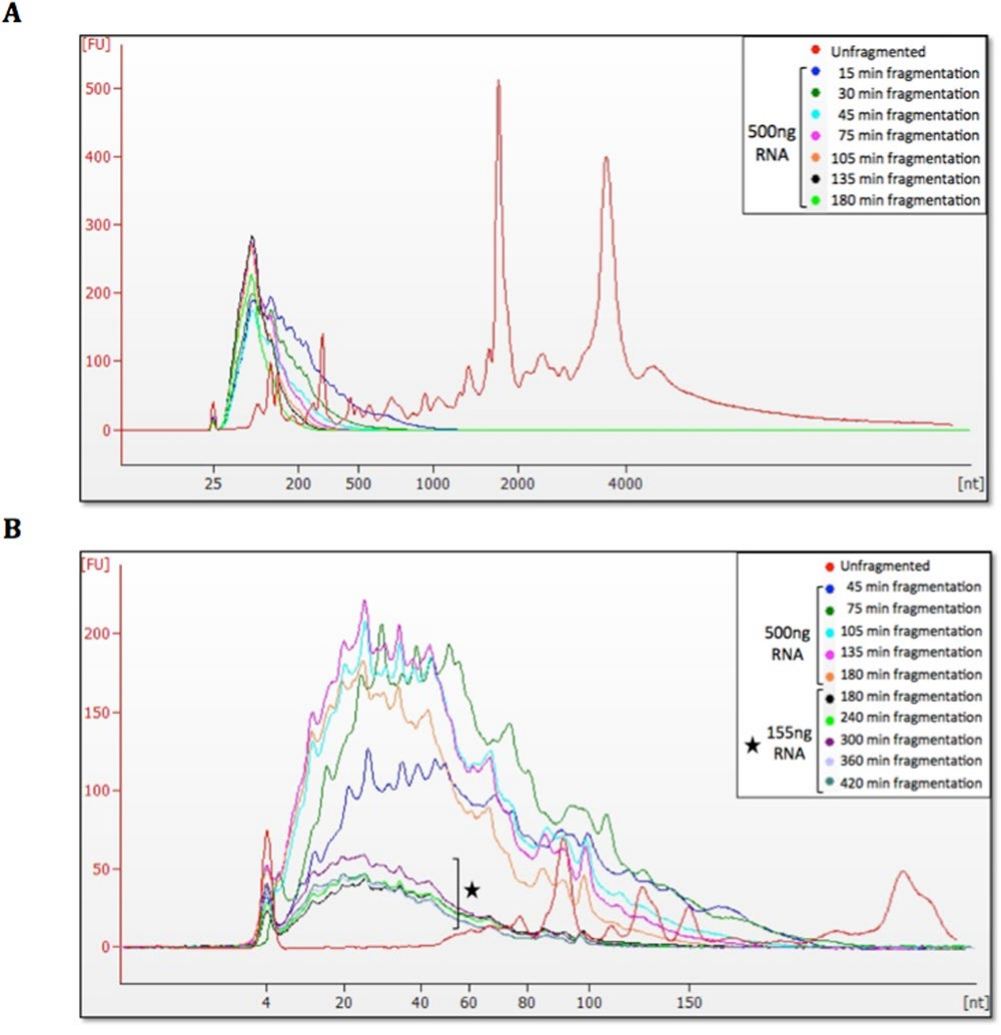
Bioanalyzer electropherogram analysis of fragmentation time-points of leukocyte RNA. Arbitrary fluorescence units (FU) are plotted as a function of RNA size in nucleotides (nt). (**A**) Analysis of fragmentation time-points 15–180 min with Agilent RNA 6000 Pico Kit shows the expected ribosomal RNA peaks in non-fragmented sample (in red), whereas after all fragmentation time-points the majority of RNAs are below 200 nt in size. (**B**) Analysis of fragmentation time-points from 45 to 420 min with Agilent Small RNA Kit shows that the majority of RNAs are smaller than 40 nt in size for all time-points, and that a plateau is reached after approximately 180 min, as no further reduction in size is observed with longer fragmentation times. For the longer fragmentation periods, reduced amounts of RNA (marked with a star) were used to better simulate the enzymatic kinetics in the presence of less (but still detectable) RNA, and the plateau region is still the same.

Next, we developed a broad protocol to isolate the full spectrum of RNA cargos from the EV samples. First, we used RNA fragmentation to increase the number of molecules as a means of reducing the formation of adaptor-dimer artifacts, which consume sequencing space and it is a known major issue when limiting amounts of nucleic acids are available for NGS library construction^26^. As EV-derived RNA obtained from plasma cannot be generally visualized in the Bioanalyzer due to its very low amounts, the fragmentation time was optimized with RNA extracted from peripheral blood leukocytes, analyzed with the Agilent RNA 6000 Pico Kit. Results showed the expected 18S and 28S rRNA peaks in non-fragmented RNA (red traces), while RNA fragmented for 180 min (light green traces) consisted of fragments <200 nt in length (Fig. 3A). Analysis with Agilent Small RNA kit of further fragmentation time-points showed that a plateau was reached after 180 min and profiles were very similar up to 420 min, with most fragments ranging from 20 to 40 nt in length **(**Fig. 3B **)**. An initial experiment was performed with time-points from 15 to 180 min, using 500ng of RNA for each point, followed by a separate follow-up experiment (using 155ng of RNA for each point and therefore shorter peaks) covering time-points from 180 to 420 min (Fig. 3B).

### The transcriptional landscape of EVs revealed by the Total Transcriptome One-Step Protocol

rRNA was the most abundant class of RNAs in the EVs, corresponding to an average of 73% of total sequences in all samples. After filtering, an average of 1.2 million reads remained for each sample (Table 2). To account for the RNA fragmentation protocol and the miRNA size range, sequences with a length between 12–25 nt (an average of 332,549 reads/sample) were first mapped against miRBase v20 by using miRDeep2 software for miRNA annotation. Reads not mapped by miRDeep2 and reads with lengths above 25 nt were then mapped to the hg19 reference genome by using STAR^22^. We obtained the coordinates of tRNAs from GtRNAdb (gtrnadb.ucsc.edu), and subsequently used BEDTools^27^ to compare the genomic coordinates to find reads overlapping with tRNA. After annotating miRNAs and tRNAs, remaining reads representing other RNA classes contained in the Ensembl database were annotated according to biotypes defined in the Ensembl glossary (http://grch37.ensembl.org/info/website/glossary.html) as depicted in Table 3 and Fig. 1. Table 3 lists the number of different transcripts identified in each RNA biotype, considering only those transcripts identified by at least two reads. Protein coding was the most diverse category in EVs, followed by short noncoding RNAs.

**Table 2:** Summary of sequencing analysis of the total transcriptome of EVs isolated from the plasma of healthy controls.

1	2	3	4	5	6	7	8	9
Sample	Raw reads	Reads after FastQ screen filter	% of filtered reads	% of rRNA filtered reads	Reads 12–25 nt used for miRNA analysis (%)	miRBase mapped reads (%)	Reads used for other RNA analysis (%)	Ensembl mapped reads (%)
C1	18,085,235	1,653,832	90.9	75.9	458,186 (27.7)	33,157 (7.2)	1,620,675 (98.0)	576,866 (35.6)
C3	7,843,315	727,633	90.7	73.4	221,985 (30.5)	23,594 (10.6)	704,039 (96.8)	170,592 (24.2)
C13	13,830,831	1,148,352	91.7	74.8	291,302 (25.4)	14,734 (5.1)	1,133,618 (98.7)	358,268 (31.6)
C15	10,111,670	1,023,795	89.9	73.8	300,569 (29.4)	25,465 (8.5)	998,330 (97.5)	232,061 (23.2)
C16	10,936,417	1,509,424	86.2	69.3	390,705 (25.9)	40,801 (10.4)	1,468,623 (97.3)	526,181(35.8)
Average	12,161,494	1,212,607	89.9	73.4	332,549 (27.8)	27,550 (8.4)	1,185,057 (97.7)	372,793 (30.1)

The % in columns 4 and 5 are relative to column 2; the % in columns 6 and 8 are relative to column 3; the % in column 7 is relative to column 6, and the % in column 9 is relative to column 8.

**Table 3:** Summary of the total number of distinct transcripts identified for different RNA categories in EVs isolated from the plasma of healthy controls.

Sample	Protein coding	Pseudogene	Long noncoding	Short noncoding			
				miRNA mature (miRBase)	tRNA (GtRNAdb)	misc_RNA	other*
C1	7,824	91	220	765			
				181	220	218	146
C3	5,461	34	101	554			
				163	172	110	109
C13	7,937	83	207	738			
				157	259	189	133
C15	6,134	44	131	626			
				162	217	133	114
C16	8,660	171	211	887			
				216	235	254	182
Average	7,203	85	174	714			
				176	221	181	137

*The category ‘other’ includes: miRNA precursors (miRBase), miRNA (Ensembl), Mt_rRNA, Mt_tRNA, rRNA, snoRNA, snRNA.

**Figure 4:**
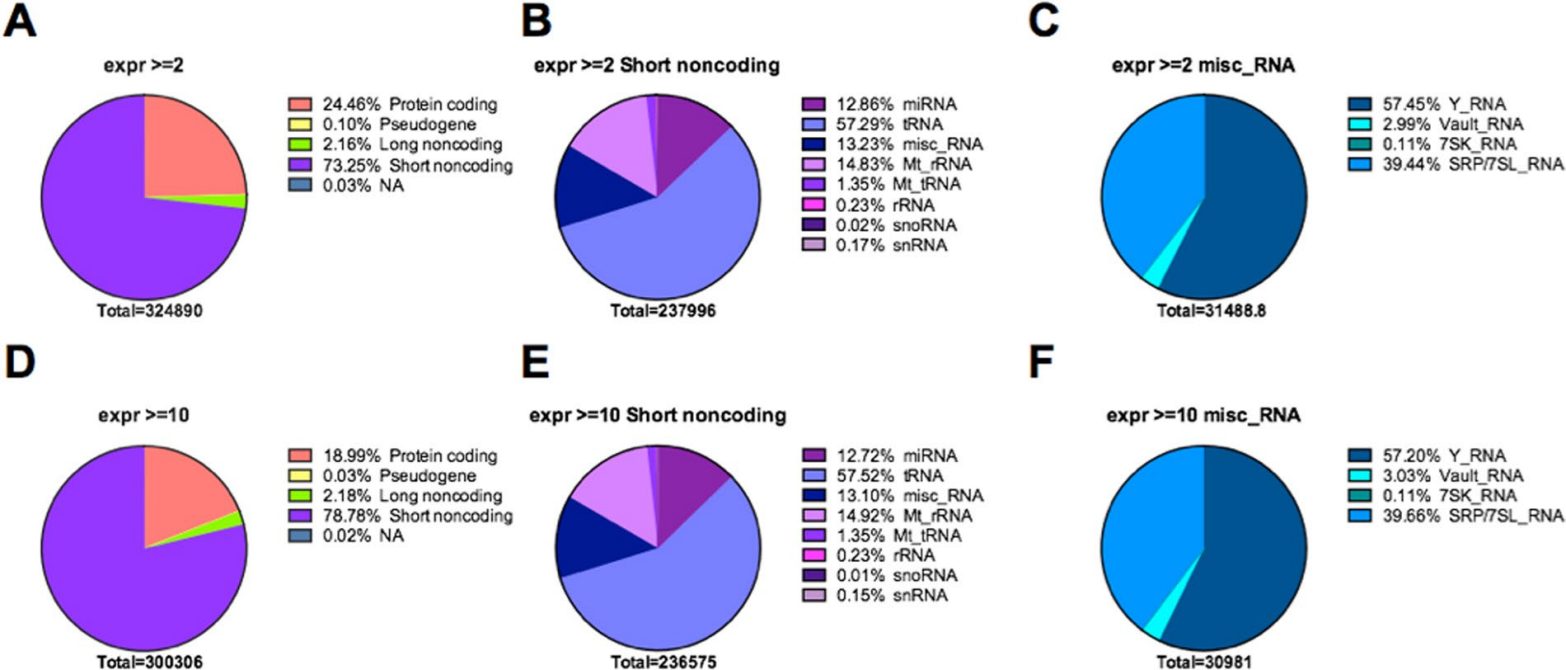
Distribution of the RNA classes identified in the sequencing analysis of the total transcriptome of EVs isolated from the plasma of healthy controls (average values for five samples). Pie charts show the distribution of mapped reads according to gene biotype, as defined by Ensembl, and sequencing coverage. Distribution of reads is shown for the four major Ensembl biotypes (**A**,**D**); the short noncoding biotype (**B**,**E**) and the misc_RNA biotype (**C**,**F**). Genes considered were covered by at least 2 reads (**A**–**C**), or by a minimum of 10 reads (**D**–**F**).

Considering relative abundances based on the number of reads mapping to each RNA category, short noncoding RNA was the most represented biotype, corresponding to 73.25% of the mapped reads. The second most represented biotype was protein-coding RNAs, represented by 24.46% of the total mapped reads, followed by long noncoding RNAs with 2.16% and pseudogenes with 0.10% of reads (Fig. 4A). In the short noncoding RNA category, tRNAs were predominant, with 57.29% of mapped reads considering short RNAs identified by at least two reads, followed by mitochondrial rRNAs (Mt_rRNA) with 14.83%, miscellaneous RNAs (misc_RNA) with 13.23%, and miRNAs with 12.86% of mapped reads (Fig. 4B). Among the misc_RNAs, Y_RNAs and SRP_7SL_RNAs were predominant, with 57.45% and 39.44% of mapped reads, followed by Vault_RNAs and 7SK_RNAs with 2.99% and 0.11% of mapped reads, respectively (Fig. 4C). Miscellaneous RNAs have been previously described as enriched in EVs^11,14^, and much remains to be understood about their regulatory functions. Similar distributions of RNA biotypes are also observed when analyses were performed with the more abundant transcripts represented by at least 10 reads (Fig. 4D–F), suggesting that our libraries, even with modest coverage after the rRNAs were filtered out, equally represented the distinct transcript classes.

For the analysis of reads mapping against longer transcripts (not miRNAs annotated by miRBase or tRNAs annotated by GtRNAdb), we implemented an additional filter to reduce potentially unreliable mapping due to short read length, which reflects mapped transcripts with poor and heterogeneous horizontal coverage. We used the Gini normalization coefficient, a measure of statistical dispersion often used to describe inequalities between parts, such as income distributions in a population^23^. In this analysis, all transcripts were divided in 10 equal parts independent of transcript size, and the Gini normalization coefficient was computed based on the total amount of reads aligning in each part. A Gini coefficient close to one indicates high inequality among the parts, which in our scenario translates to heterogeneous transcript coverage, suggesting alignment issues. After manual curation, considering the sequencing coverage obtained here, we elected a maximum Gini coefficient of 0.6 as a cut-off value based on the correlation of adequate horizontal coverage.

In Suppl. Table 2 we list the average expression levels for transcripts annotated in the Ensembl database, after excluding miRNAs annotated by miRBase and tRNAs annotated by GtRNAdb, and that passed the Gini coefficient filtering step, normalized to 100,000 reads/sample relative to the total counts of all mapped Ensembl transcripts. It can be observed that most of these (80.1%) were present in all five individuals and should, therefore, consist in the core EV-transcriptome.

Notably, in addition to the RNA classes described above, our sequencing approach also enables the identification and quantification of backspliced circular RNAs (circRNAs), which have been recently identified in EVs^28,29^. After considering only the predicted RNA-circularization events that were confirmed by at least two reads, we found a total of 83 different putative circRNAs in EVs isolated from the plasma of healthy women, including 68/83 (82%) that are also present with the same genomic coordinates (start and end) in circBase^30–34^ and 15 novel circRNA structures (Suppl. Table 3). Representative circRNAs were validated by PCR (by using outward primers to cover the circularizing point), followed by Sanger DNA sequencing (Suppl. Table 3, see an example in Suppl. Figure 1).

### Confirmation of differential miRNA abundance and intravesicular location of transcripts

**Figure 5:**
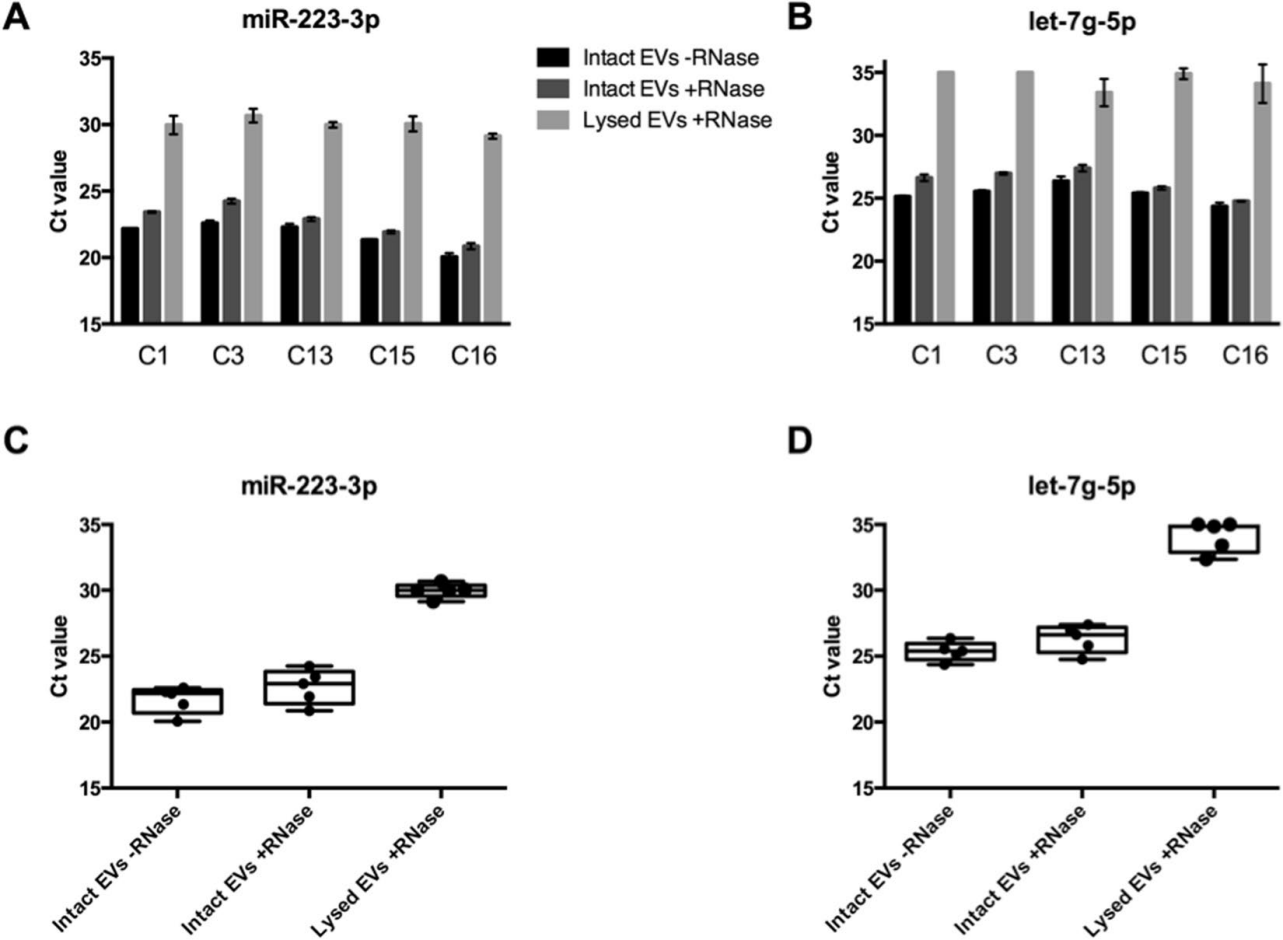
Quantitative RT-PCR analysis of two miRNAs (miR-223-3p and let-7g-5p) identified by sequencing. The treatment of intact EVs with RNase before RNA extraction minimally altered Ct values of both miRNAs, strongly suggesting that these miRNAs were derived from the EVs-cargo and thereby protected from digestion by the membrane bilayer, whereas there was a large increase in Ct values when lysed EVs were similarly treated. Undetermined values (no detection by qRT-PCR) were assigned as Ct = 35. (**A**,**B**) Bar-graphs for miR-223-3p and let-7g-5p, showing Ct values for each treatment per sample; (**C**,**D**) Box-plots for miR-223-3p and let-7g-5p, showing Ct values per treatment (dots are results from each one of the five samples).

In order to verify the quantitative capabilities of our NGS-based protocol, we next selected one miRNA with high read counts (hsa-miR-223-3p - 313 reads/million) and one with low read counts (hsa-let-7g-5p - 19 reads/million). The expression value in reads/million was calculated by taking the number of reads mapped for each miRNA, multiplying by 1,000,000 and dividing by the total number of reads for that particular sample. This normalization coefficient was calculated for each sample and an average of the five samples is reported above. Quantitative RT-PCR validation of these miRNAs showed a ~12-fold difference in quantity calculated by 2^(delta Ct), comparable to a differential expression of ~16-fold calculated by NGS. At the same time, we conducted an experiment to confirm the intravesicular location of these same molecules by treating the EVs with RNase-A before RNA extraction. Three conditions were compared in parallel, starting with the same plasma volumes (0.5 ml) for each sample: i) mock-treatment of intact EVs, ii) intact EVs treated with RNase-A, and iii) disrupted EVs treated with RNase-A. Whereas the Ct values for intact EVs – mock- and RNase A-treated – were quite similar (target reduction varying from 1.32-fold to 3.13-fold after RNAse treatment), the treatment of lysed EVs showed a drastic Ct increment, corresponding to a 64.3-525.8-fold reduction in miRNA quantities when compared to intact EVs, strongly suggesting that most RNAs represented in our protocol are indeed intra-vesicular (Fig. 5).

## Discussion

Extracellular vesicle analysis is an essential component of liquid biopsies in contemporary research and medical applications. Thus, the dissection of EV-cargo from human samples and animal models has the potential to reveal elements that may be key for tissue homeostasis and tissue-tissue communication. In cancer patients, the information provided by plasma-derived EVs may contain valuable biomarkers for precision medicine, complementing the value of other biomarkers found in circulating tumor cells or circulating tumor DNA, including molecules that can play a role in priming tissues that will receive metastatic cells^35^. In this sense, the capability of defining the total EV transcriptome is remarkably relevant.

Here we present a new approach: the One-Step Total Transcriptome Protocol, which, for the first time, allowed the description of all known biotypes of plasma derived EV-RNAs in a single NGS library. A major technical hurdle found during NGS analysis of limited RNA input samples is the formation of an excess of adaptor-dimers during library preparation, a technical issue that is present when the available amount of RNA is <200 ng^26,36^. In this sense, our protocol of RNA fragmentation by RNase-III treatment allowed the increment of the number of molecules available for ligation to adaptors, with no need to amplify the initial material. This feature counteracted the artifactual formation of adaptor-dimers and avoided possible amplification and NGS-representation biases of the heterogeneous vesicular transcriptome.

Previous results from our group (data not shown) also obtained from human plasma-derived EVs isolated with this same ultracentrifugation protocol, but using no fragmentation step, resulted in 46–82% of the reads to be adaptor-dimers, having as input the total RNA isolated from 5 ml of plasma-derived EVs. After the adoption of the protocol introduced here, the frequency of adaptor-dimers was reduced to 13–38%, by using RNA from 2.5 ml of plasma. The enzymatic digestion of the whole RNA-content of EVs, reducing all transcripts to a size range of small RNAs but still permitting their precise annotation and quantification, allowed us to optimize the library preparation protocol intended for small RNAs, and yet to represent other RNAs subtypes, and to ultimately cover essentially the whole EV-transcriptome with a single library.

This protocol revealed the presence of thousands of transcripts from distinct RNA classes inside the vesicles, including long RNAs and circRNAs. The fragmentation of these molecules, with an original average size of 2.5–3.5Kb, to an average size of 38 nt allowed us to increase the number of these molecules at least 65-fold, negatively impacting the formation of undesirable adaptor-dimers. Besides reducing the amounts of adaptor-dimers, the benefits of this approach include i) the reduction of reagents amount, time and sequencing costs to less than half, ii) the ability to use low sample volume input and iii) the unbiased analysis of the entire transcriptome at once, without the inherent prejudice of methodologies based on size selection or template pre-amplification. In this sense, it is well worth mentioning that a recent large-scale RNA-seq study showed a clear batch effect in the percentage distribution of RNA species identified due to variations in the gel size selection step – 40 gels were run during the library preparation of 192 samples and the relative quantities of miRNAs subsequently identified were consistently higher in the first 19 gels compared to the remaining 21 gels, where more piwiRNAs were identified^12^.

Another limitation that may derive from the fragmentation of the molecules is the sometimes-ambiguous mapping of some reads to mRNAs. However, this matter was solved with a more stringent bioinformatics pipeline for mapping short reads, incorporating the Gini normalization coefficient as a means to indicate transcripts with good and homogeneous horizontal coverage as an extra step to essentially eliminate ambiguous mapping. Moreover, the Gini coefficient would be important to be implemented in further analysis where the evaluation of differential expression among groups of samples is a goal of the study.

For each sample of the study we generated an average of 12.1 million reads (Table 2). After filtering with FastQ screen, about 73% of the EVs transcriptome showed to be derived from human rRNA genes. The field has been somewhat controversial regarding the presence or absence of rRNA in EVs, with several studies claiming their absence due to the lack of the characteristic 18S and 28S rRNA peaks in Bioanalyzer traces^3,7,37,38^. However, these peaks only reflect the presence of intact rRNA molecules and recent whole transcriptome RNA-seq studies (similar to ours) have found up to 97% of rRNA and indeed showed that EVs are enriched in rRNAs fragments, which are not visible in Bioanalyzer profiles^16^. One might emphasize that for clinical patient-derived samples, as of today, the use of rRNA depletion kits is usually not feasible due to the requirement of high amounts of starting RNA mass (>100 ng), which are far beyond the RNA-content available in typical clinical samples (<1 ng). RNA-seq studies that performed size selection procedures (either gel- or bead-based) might perhaps be able to circumvent the rRNAs detection by enriching for a smaller RNA fraction. However, this would likely lead to a biased view of the EVs-cargo, as a substantial population of mRNAs and long noncoding RNAs is bound to be also excluded together with the rRNA sequences, due to an overlap in transcript length.

The transcriptional landscape of circulating EVs in these subjects revealed that the majority of sequences, besides the rRNAs, correspond to short noncoding RNAs. In this category, the major players are tRNAs, miscRNAs and miRNAs. Among the miscRNAs found in EVs, we highlight the abundance of Y-RNAs, noncoding molecules that associate with replicating euchromatin^39^, have been associated with breast cancer^40^, atherosclerosis-related diseases^41^ and are enriched in EVs^11,14^ (Fig. 4). Among the top-50 most abundant transcripts identified in EVs (Suppl. Table 2), 38% are not described in EV-cargo databases^42,43^, which do not cover lncRNAs. Notably, Vesiclepedia^42^ has only seven studies with RNA analysis of plasma/serum samples, and none of these employed NGS technology for EVs characterization. Also, only two out of the seven studies analyzed mRNA profiles, all other studies focused solely on miRNAs.

Our analysis also confirmed the presence of back-spliced circRNAs in EVs. These transcripts - characterized by the presence of some exons spliced in non-canonical order due to the circularization of an otherwise linear transcript - have been found to be stable, abundant, evolutionarily conserved and in some cases these molecules appear to act as miRNA sponges that can block miRNA-regulation of their linear counterpart-genes in recipient cells^28–30,44–46^. Our findings based in the consensus alignment of high-quality reads indicated the presence of 83 circRNAs in these individuals, including 15 new circRNAs, of which seven have the same start or end positions of previously described circRNAs present in circBase, suggesting possible new isoforms and eight that were not previously annotated (Suppl. Table 3).

Our qRT-PCR validation of the NGS results demonstrated the intra-vesicular location of the isolated RNA and suggested that their quantification by NGS, given by read-counts derived from the protocol presented here, does not introduce significant quantification biases, which is relevant for the study of potential biomarkers in pathological conditions. The protocol presented here enables for the first time the analysis of total transcriptomes when only limiting amounts of RNA are available, as in the case of extracellular vesicles, allowing the representation of all RNA classes while also preserving the quantitative differences between molecules. As such, we conclude that this One-Step methodology might become a standard in the emerging field of liquid biopsy applications.

## Electronic supplementary material

Supplementary Information

Supplementary Table 1

Supplementary Table 2

Supplementary Table 3

## References

[1] Barteneva NS (2013). Circulating microparticles: square the circle. BMC Cell Biol.

[2] Colombo M, Raposo G, Théry C (2014). Biogenesis, Secretion, and Intercellular Interactions of Exosomes and Other Extracellular Vesicles. Annu Rev Cell Dev Biol.

[3] Valadi H (2007). Exosome-mediated transfer of mRNAs and microRNAs is a novel mechanism of genetic exchange between cells. Nat Cell Biol.

[4] Amorim M (2014). The overexpression of a single oncogene (ERBB2/HER2) alters the proteomic landscape of extracellular vesicles. Proteomics.

[5] Abels ER, Breakefield XO (2016). Introduction to Extracellular Vesicles: Biogenesis, RNA Cargo Selection, Content, Release, and Uptake. Cell Mol Neurobiol.

[6] Al-Nedawi K (2008). Intercellular transfer of the oncogenic receptor EGFRvIII by microvesicles derived from tumour cells. Nat Cell Biol.

[7] Skog J (2008). Glioblastoma microvesicles transport RNA and proteins that promote tumour growth and provide diagnostic biomarkers. Nat Cell Biol.

[8] Deregibus MC (2007). Endothelial progenitor cell derived microvesicles activate an angiogenic program in endothelial cells by a horizontal transfer of mRNA. Blood.

[9] Ahsan NA (2016). Presence of Viral RNA and Proteins in Exosomes from Cellular Clones Resistant to Rift Valley Fever Virus Infection. Front Microbiol.

[10] Huang X (2013). Characterization of human plasma-derived exosomal RNAs by deep sequencing. BMC Genomics.

[11] Nolte’T Hoen ENM (2012). Deep sequencing of RNA from immune cell-derived vesicles uncovers the selective incorporation of small non-coding RNA biotypes with potential regulatory functions. Nucleic Acids Res.

[12] Yuan T (2016). Plasma extracellular RNA profiles in healthy and cancer patients. Sci Rep.

[13] Quek, C. *et al*. Defining the purity of exosomes required for diagnostic profiling of small RNA suitable for biomarker discovery. *RNA Biol* 00–00 10.1080/15476286.2016.1270005 (2016). 10.1080/15476286.2016.1270005 PMC5324750 28005467

[14] Lefebvre FA (2016). Comparative transcriptomic analysis of human and Drosophila extracellular vesicles. Sci Rep.

[15] San Lucas FA (2016). Minimally invasive genomic and transcriptomic profiling of visceral cancers by next-generation sequencing of circulating exosomes. Ann Oncol.

[16] Jenjaroenpun P (2013). Characterization of RNA in exosomes secreted by human breast cancer cell lines using next-generation sequencing. PeerJ.

[17] Schageman, J. *et al*. The complete exosome workflow solution: From isolation to characterization of RNA cargo. *Biomed Res Int* **2013**, (2013). 10.1155/2013/253957 PMC3800616 24205503

[18] Li M (2014). Analysis of the RNA content of the exosomes derived from blood serum and urine and its potential as biomarkers. Philos Trans R Soc B Biol Sci.

[19] Théry, C. *et al*. Isolation and characterization of exosomes from cell culture supernatants and biological fluids. *Curr Protoc cell Bio*l Chapter 3, 3.22.1-3.22.29 (2006). 10.1002/0471143030.cb0322s30 18228490

[20] Andrews, S. No Title http://www.bioinformatics.babraham.ac.uk/projects/fastq_screen/ (2011). Available at: http://www.bioinformatics.babraham.ac.uk/projects/fastq_screen/.

[21] Mackowiak, S. D. Identification of novel and known miRNAs in deep-sequencing data with miRDeep2. *Curr Protoc Bioinform*a Chapter 12, Unit12.10. (2011). 10.1002/0471250953.bi1210s36 22161567

[22] Dobin A (2013). STAR: Ultrafast universal RNA-seq aligner. Bioinformatics.

[23] Gini C (1921). Measurement of Inequality of Incomes. Econ J.

[24] Glažar P, Papavasileiou P, Rajewsky N (2014). circBase: a database for circular RNAs. RNA.

[25] Sanguinetti CJ, Dias Neto E, Simpson AJ (1994). Rapid silver staining and recovery of PCR products separated on polyacrylamide gels. Biotechniques.

[26] Head, S. R. *et al*. Library construction for next-generation sequencing: overviews and challenges. *Biotechniques* **56**, 61–4, 66, 68, passim (2014). 10.2144/000114133 PMC4351865 24502796

[27] Quinlan AR, Hall IM (2010). BEDTools: A flexible suite of utilities for comparing genomic features. Bioinformatics.

[28] Li Y (2015). Circular RNA is enriched and stable in exosomes: a promising biomarker for cancer diagnosis. Cell Res.

[29] Lasda E, Parker R (2016). Circular RNAs co-precipitate with extracellular vesicles: A possible mechanism for circrna clearance. PLoS One.

[30] Jeck WR (2013). Circular RNAs are abundant, conserved, and associated with ALU repeats. RNA.

[31] Memczak S (2013). Circular RNAs are a large class of animal RNAs with regulatory potency. Nature.

[32] Salzman J, Chen RE, Olsen MN, Wang PL, Brown PO (2013). Cell-type specific features of circular RNA expression. PLoS Genet.

[33] Zhang Y (2013). Circular intronic long noncoding RNAs. Mol Cell.

[34] Rybak-Wolf A (2015). Circular RNAs in the Mammalian Brain Are Highly Abundant, Conserved, and Dynamically Expressed. Mol Cell.

[35] Peinado H (2017). Pre-metastatic niches: organ-specific homes for metastases. Nat Rev Cancer.

[36] Buschmann D (2016). Toward reliable biomarker signatures in the age of liquid biopsies - how to standardize the small RNA-Seq workflow. Nucleic Acids Res.

[37] Cheng L, Sharples RA, Scicluna BJ, Hill AF (2014). Exosomes provide a protective and enriched source of miRNA for biomarker profiling compared to intracellular and cell-free blood. J Extracell vesicles.

[38] Ji H (2014). Deep Sequencing of RNA from Three Different Extracellular Vesicle (EV) Subtypes Released from the Human LIM1863 Colon Cancer Cell Line Uncovers Distinct Mirna-Enrichment Signatures. PLoS One.

[39] Kheir E, Krude T (2017). Non-coding Y RNAs associate with early replicating euchromatin in concordance with the origin recognition complex. J Cell Sci.

[40] Dhahbi JM, Spindler SR, Atamna H, Boffelli D, Martin DI (2014). Deep Sequencing of Serum Small RNAs Identifies Patterns of 5′ tRNA Half and YRNA Fragment Expression Associated with Breast Cancer. Biomark Cancer.

[41] Repetto E (2015). RNY-derived small RNAs as a signature of coronary artery disease. BMC Med.

[42] Kalra H (2012). Vesiclepedia: a compendium for extracellular vesicles with continuous community annotation. PLoS Biol.

[43] Kim D-K (2013). EVpedia: an integrated database of high-throughput data for systemic analyses of extracellular vesicles. J Extracell vesicles.

[44] Hansen TB (2013). Natural RNA circles function as efficient microRNA sponges. Nature.

[45] Salzman J, Gawad C, Wang PL, Lacayo N, Brown PO (2012). Circular RNAs are the predominant transcript isoform from hundreds of human genes in diverse cell types. PLoS One.

[46] Piwecka, M. *et al*. Loss of a mammalian circular RNA locus causes miRNA deregulation and affects brain function. *Science*10.1126/science.aam8526 (2017). 10.1126/science.aam8526 28798046

