# Supplementary material for: A total transcriptome profiling method for plasma-derived extracellular vesicles: applications for liquid biopsies
Source: Sci Rep. 2017 Oct 31;7:14395. doi: 10.1038/s41598-017-14264-5 (PMC5663969; doi:10.1038/s41598-017-14264-5)
Supplement: Supplementary file 1 — Supplementary Information [file 41598_2017_14264_MOESM1_ESM.doc]

**SUPPLEMENTARY INFORMATION**

**A total transcriptome profiling method for plasma-derived extracellular vesicles: applications for liquid biopsies**

Maria G. Amorim^1^, Renan Valieris^2^, Rodrigo Drummond^2^, Melissa P. Pizzi^1^, Vanessa Freitas^3^, Rita Sinigaglia-Coimbra^4^, George A. Calin^5^, Renata Pasqualini^6^, Wadih Arap^6^, Israel T. Silva^2,7^, Emmanuel Dias-Neto^1,8*^, Diana N. Nunes^1*^


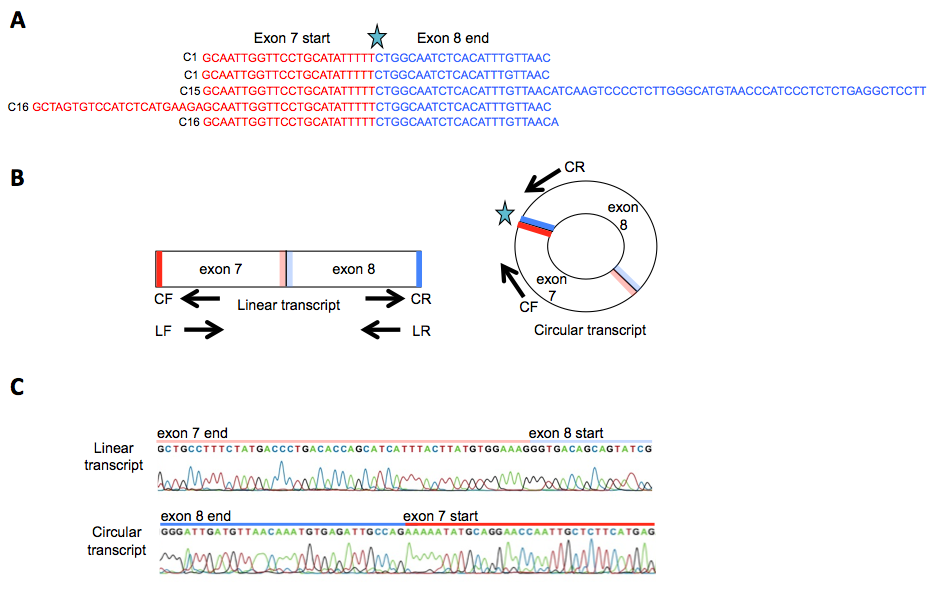


**Supplementary Figure 1 – Validation of a circRNA in CORO1C gene.**

**A)** Sequencing reads identifying circRNA (2 reads in sample C1, 1 read in sample C15, and 2 reads in sample C16). The blue star represents the junction of the start of exon 7 (shown in red) with the end of exon 8 (shown in blue). **B)** Schematic of the PCR products generated with inward-facing primers (LF and LR) that amplify linear transcript, and outward-facing primers (CF and CR) that amplify circular transcript. Arrows represent the location of the primers. The dark red line represents the start of exon 7, the light red line the end of exon 7, the light blue line the start of exon 8 and the dark blue line the end of exon 8. **C)** Sanger sequencing of the PCR products showing the linear and circular transcripts.


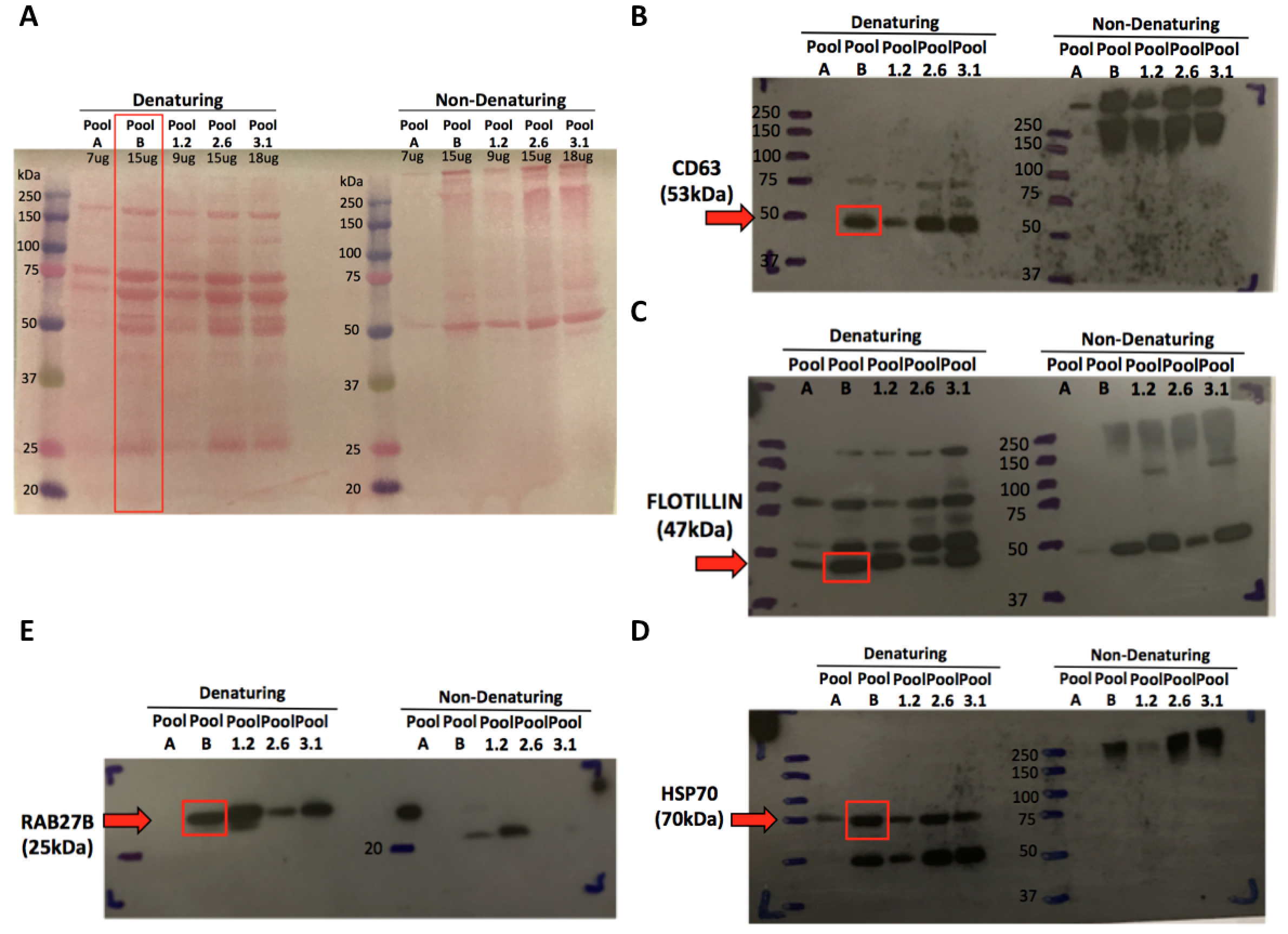


**Supplementary Figure 2 – Uncropped images of blots shown in Figure 2C (indicated with red box).**

**A)** Ponceau staining of membrane after transfer. Before blocking, the membrane was cut right below the 37kDa band, into top and bottom membranes. **B)** Blot of top membrane after incubation with CD63 antibody. **C)** Blot of same top membrane (post-stripping) after incubation with FLOTILLIN antibody. **D)** Blot of same top membrane (post-stripping) after incubation with HSP70 antibody. **E)** Blot of bottom membrane (post-stripping) after incubation with RAB27B antibody.


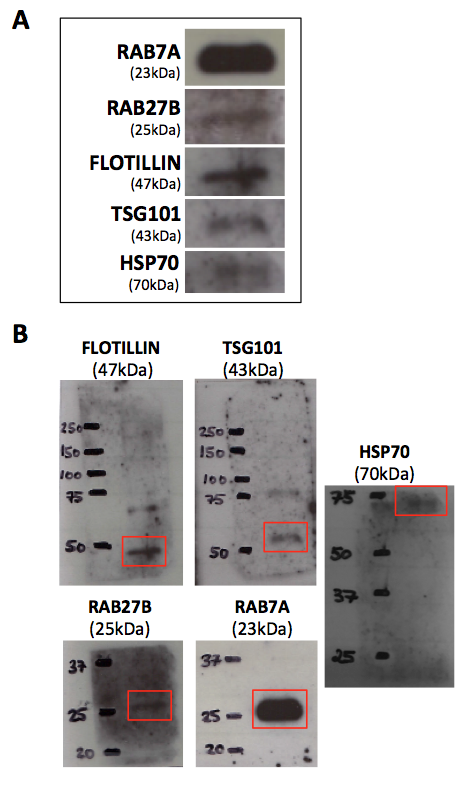


**Supplementary Figure 3 –** Western blot of the vesicle-associated markers RAB7A, RAB27B FLOTILLIN, TSG101, and HSP70 from a pool of different plasma samples (n=4) as that shown in Fig 2C. **A)** Cropped images. **B)** Uncropped blots. The same lysate was applied to three lanes, and the membranes were cut before blocking and antibody incubation.

**Supplementary Table Legends:**

**Supplementary Table 1** – Read counts of RNAs annotated in each sample evaluated. The first tab summarizes the data for all samples (n=5) considering RNAs identified by distinct levels of coverage (≥2 or ≥10 reads - percentages were calculated by considering the sum of annotated reads per biotype relative to the total sum of annotated reads) and subsequently there are two tabs for each sample: one with all biotypes and another with only the misc_RNA category.

**Supplementary Table 2** – Average expression levels of transcripts annotated in Ensembl database, after excluding miRNAs annotated by miRBase and tRNAs annotated by GtRNAdb, for the five evaluated samples. Data were filtered by the Gini coefficient and normalized to 100,000 reads relative to the total counts of all mapped Ensembl transcripts per sample.

**Supplementary Table 3** – Predicted RNA-circularization events by find_circ (https://omictools.com/find-circ-tool ) and confirmed by ≥2 reads.
